# Point of care ultrasonography in the pediatric emergency department

**DOI:** 10.1186/s13052-018-0520-y

**Published:** 2018-07-27

**Authors:** Julien Le Coz, Silvia Orlandini, Luigi Titomanlio, Victoria Elisa Rinaldi

**Affiliations:** 1 Department of Pediatric Emergency Care, APHP - Hopital Robert Debré, 75019 Paris, France; 2 Department of Pediatrics, Ospedale della Donna e del Bambino, University of Verona, 37126 Verona, Italy; 3 Sorbonne Paris Cité, INSERM U1141, DHU Protect, Paris Diderot University, 75019 Paris, France; 4 Pediatric Migraine and Neurovascular Diseases Unit, APHP - Hopital Robert Debré, 75019 Paris, France; 5 Pediatric Emergency Department, INSERM U1141 – Developmental Neurobiology & Neuroprotection, Paris Diderot -Sorbonne-Paris Cité University, Robert Debré Hospital, 48 Boulevard Serurier, 75019 Paris, France

**Keywords:** POCUS, PED, FAST, Ultrasound, Paediatrics

## Abstract

**Importance:**

Point-of-care ultrasonography (POCUS) allows to obtain real-time images to correlate with the patient’s presenting signs and symptoms. It can be used by various specialties and may be broadly divided into diagnostic and procedural applications.

**Objective:**

We aimed at reviewing current knowledge on the use of POCUS in Pediatric Emergency Departments (PEDs).

**Findings:**

US diagnostic capacity in paediatric patients with suspected pneumonia has been studied and debated whereas literature regarding the usefulness of point-of-care echocardiography in the pediatric setting is still limited. Similarly, Focused Assessment with Sonography for Trauma (FAST) has become a standard procedure in adult emergency medicine but it is still not well codified in the pediatric practice. Concerning procedural applications of POCUS we identified 4 main groups: peripheral vascular access, bladder catheterizations, identification and drainage of abnormal fluid collections and foreign body identification.

**Conclusions and relevance:**

Bedside emergency ultrasound is routinely used by adult emergency physicians and in the last 10 years its application is recognized and applied in PED. Pediatric emergency physicians are encouraged to familiarize with POCUS as it is a safe technology and can be extremely helpful in performing diagnosis, managing critical situations and guiding procedures, which results in globally improving pediatric patients care.

## Introduction

Point-of-care ultrasonography (POCUS) is defined as ultrasonography brought to the patient and performed by the clinician in real time [1]. With this technique, ultrasound (US) images can be obtained immediately and the physician can correlate real-time images with the patient’s presenting signs and symptoms [2]. POCUS is easily repeatable if the patient’s conditions change, it can be used by various specialties in different situations and may be broadly divided into diagnostic and procedural applications. The premise of a POCUS examination is a focused examination in answer to a specific clinical question [3]. In particular the American Academy of Pediatrics policy statement cautions that “clinicians should be aware that point of care ultrasonography is better used as a rule in and not a rule out diagnostic modality” [4].

## Methods

We aimed at reviewing current knowledge on the most recognized fields of application of ultrasound and point of care ultrasonography in Pediatric Accidents and Emergency Departments. To identify articles a literature search was carried out in PubMed and Embase for most recent studies published until August 31, 2017. The keywords searched for were “ultrasound”, “point of care ultrasound”, “POCUS”, “children”, “pediatrics” and “emergency department”. Main topics of interest were subdivided in diagnostic applications (cardiopulmonary, abdominal, obstetrics and gynecology, traumatology and musculoskeletal emergencies) and procedural applications (vascular access, drainage of fluid collections and foreign body identification). The paper was structured as a narrative review. Studies and case reports were included if they focused on the effective application of POCUS performed by pediatric emergency physicians in pediatric emergency departments with evidence in good clinical practice such as earlier diagnosis or reduced patient’s length of stay. Papers published in peer reviewed journals, quoted as recurrent references and written in English as the worldwide recognized scientific language, were selected. We excluded studies concerning POCUS performed by radiologists, used in non-emergency situations or in different fields of application /off topics application. Full articles reviews, metanalysis and clinical trials were preferred to single case reports.

## Results

### Diagnostic applications of US in Accidents & Emergencies

Pediatric emergency medicine diagnostic applications can be subdivided mainly in 5 groups: cardiopulmonary, abdominal, obstetrics and gynecology, traumatology, and musculoskeletal.

1. ***Pulmonary and cardiac emergencies***

Recent literature has highlighted US diagnostic capacity compared to radiography in paediatric patients with suspected pneumonia [5]. In particular, a recent metanalysis that included 8 studies (2 on neonates and 6 on children with suspected pneumonia) for a total of 765 patients, revealed that lung US had a high global sensitivity 96% (95% CI: 94–97%) and specificity 93% (95% CI 90–95,7%) for the diagnosis of pneumonia [5].

Moreover, Basile et al. in an observational cohort study including 106 infants with bronchiolitis found that lung ultrasound findings correlated with the clinical evaluations permitting a precocious identification of infants who were in need of supplementary oxygen with a high specificity (98.7%), and sensitivity (96.6%) [6].

However, limitations of lung US consist in the fact that the pulmonary parenchyma is studied indirectly through artefacts. Moreover, in the case of detecting pneumonia, this technique cannot exclude anomalies that don’t reach the pleurae. This is particularly important in the process of “ruling out” a pulmonary infection since some consolidations are medial and surrounded by aerated parenchyma which prevents visualization.

Bedside echocardiography is part of the FAST examination and can be performed in several clinical contexts in the pediatric emergency department (PED), including chest trauma, chest pain, dyspnea, tachycardia, hypotension/shock and cardiac arrest.

However, literature regarding the use of point-of-care echocardiography (POCE) in a pediatric setting is limited and most of the data are derived from case reports.

The detection of pericardial effusion and tamponade, the evaluation of the global contractility, the assessment of left ventricular function and right ventricular filling, the assessment of Pulseless Electrical Activity and asystole [7–11] are the most common indications for POCE. Longjohn et al. demonstrated in a prospective observational study that pediatric emergency physicians could be able to perform POCE as effectively as a pediatric cardiologist in the PED, with an accurate assessment of left ventricle (LV) systolic dysfunction, cardiac preload, and presence or absence of pericardial effusion [12]. In their study the overall sensitivity and specificity of POCE compared to a formal echocardiogram was respectively 95% (95% CI, 82–99%) and 83% (95% CI, 64–93%) with a strong interobserver agreement (kappa range: 0.73–0.87) across all three outcomes. In 3 case reports described by Milner et al. concerning children with an altered mental status and tachycardia, the cardiac ultrasound evaluation was performed by a cardiologist and authors stated that if POCE had been performed by trained pediatric emergency physicians, the diagnosis would have been made earlier with consequent improved outcomes [13]. Similarly, Smith et al. described a case where pericardial effusion with tamponade was successfully diagnosed in a 12 years old boy with POCE performed by pediatric emergency physicians [14].

Other applications of POCE could have an interest for pediatric emergency physicians in the future. Cheng et al. suggest that POCE can be useful for the diagnosis and the management of infective endocarditis [15]. Presley et al. present a case of bilateral pulmonary embolisms in an adolescent patient to illustrate the benefits of a timely diagnosis of right ventricular dysfunction by point-of- care echocardiography performed by emergency medicine physicians [16].

2) ***Abdominal US***

Abdominal US can facilitate the diagnosis of a wide-range of bowel pathological conditions but its use as point-of-care US performed by pediatric emergency physicians is still limited. Acute appendicitis (AA) is the most common surgical emergency in children and trained pediatric emergency physicians are able to confirm their diagnosis through POCUS with a 60–96% sensitivity and 68–98% specificity [17]. US has been used for confirming the diagnosis of acute appendicitis since 1981 [18]. POCUS performed by trained pediatric emergency physicians can be crucial in reducing time to diagnosis and definitive treatment, decreasing morbidity and prolonged hospital stay [19–21]. However, a negative POCUS is not sufficient to rule out AA and further investigations are recommended to perform this diagnosis [22, 23].

In some case reports pediatric emergency physicians are able to accurately perform abdominal POCUS and rapidly obtain information about ileus and small bowel obstruction, pneumoperitoneum, diverticulitis, abdominal wall masses and suspected hernias [24–28]. Moreover, US is the first-choice diagnostic test in the evaluation of children with suspected intussusception and pyloric stenosis with an overlap sensitivity and specificity when performed by imaging specialists, junior residents or pediatric emergency physicians [29]. POCUS can rule in the diagnosis of intussusception and could be useful in the estimation of blood flow, in the identification of free fluid or echogenic foci that could suggest a failure in intussusception reduction or complications [17, 30]. Concerning pyloric stenosis, frequently suspected in projectile vomiting, there is no currently definitive consensus regarding absolute measurements [29] and ulterior research is needed in this field.

3) ***Obstetric and gynecologic emergencies***

The main application for POCUS in the obstetric and gynecologic field is to distinguish between normal pregnancy and ectopic pregnancy in a female adolescent patient complaining of abdominal pain [17, 29, 31].

In pregnant patients, POCUS performed by pediatric emergency physicians can diagnose intrauterine or ectopic pregnancy with a demonstrated 100% sensitivity and 95% specificity, and a significantly reduced time in diagnosis and treatment of patients with ruptured ectopic pregnancy [31]. On the other hand, US evaluation of ovarian torsion, ovarian cyst and tubo-ovarian abscess still requires advanced skills and experience.

4) ***Traumatology: Eco FAST in trauma and shock***

“Focused Assessment with Sonography for Trauma,” with the acronym FAST, is a term coined at an International Consensus Conference in 1997 to describe the most common application of US in the initial evaluation of the trauma patient [32]. During this International Consensus it was defined as a real-time sonographic scanning in four distinct regions of the torso (the four Ps): pericardial, perihepatic, perisplenic, and pelvic [32]. The extended FAST (e-FAST) also includes examination of the chest for pneumothorax [33]. E-FAST is therefore used for the detection of free intraperitoneal fluid, free fluid in the pelvis, pericardial fluid, pleural effusion, and pneumothorax [1]. The thorax is evaluated for fluid at the flanks and for pneumothorax anteriorly, whereas the presence of peritoneal fluid is assessed with views of the hepatorenal space, splenorenal space, and retrovesicular spaces [1]. Whereas e-FAST has become a standard procedure in adult emergency medicine, it is still not well codified in the pediatric practice. Although it can be performed in 3 to 5 min, it is non-invasive, portable, it can be performed during resuscitation and it does not offer a radiation dose, FAST has not yet been embraced with enthusiasm in all pediatric trauma centers [34]. This is probably due to the fact that in the past it’s sensitivity has been characterized as low as 45 to 55% [35], mostly because nearly 40% of abdominal injuries in children are reported not to be associated with free fluid [36]. Nevertheless a 20% gain in predictive positive value had been already reported with the association of clinical examination and ultrasound (88%) versus simple clinical examination (69%) [37].

In 2007, Holmes et al. reviewed the best estimates of abdominal ultrasonography performance revealing a sensitivity of 80% (95% confidence interval CI 76–84%), a specificity of 96% (95% CI 95–97%) a positive likelihood ratio of 22.9 (95% CI 17.2–30.5) and a negative likelihood ratio of 0.2 (95% CI 0.16–0.25) in identifying children with hemoperitoneum [38]. Therefore, a negative e-FAST can be questionable in ruling out the presence of an intrabdominal injury, whereas the detection of free intraperitoneal fluid in a stable child should be followed by other diagnostic examinations such as abdominal computed tomographic scanning [38].

However, although CT provides detailed information about internal injuries, it exposes children to high radiation dosages with a consequent increased risk of future malignancy. For this reason, similarly to their previous work for identifying children at a low risk of serious brain injury after head trauma [39], the Pediatric Emergency Care Applied Research Network (PECARN) derived a clinical prediction rule for children with blunt torso and abdomen trauma to identify those at a high risk and at a low and for intra-abdominal injury [40]. In this multicenter study, a 7-point decision and prediction rule were derived only from clinical data, to identify children with a serious intra-abdominal injury requiring ulterior examinations or acute intervention. In particular, according to these authors, the risk of abdominal injury in those patients with lower risk clinical variables was approximately less than 1%. Therefore, because FAST examination has been reported to have a negative likelihood ratio of 0.2 [38], a normal FAST examination in those patients with a low pre-FAST risk for intrabdominal injury indicates such a low risk for intrabdominal injury that CT can be considered unnecessary [40]. Overall the use of the FAST examination has been shown to reduce the need for CT or diagnostic peritoneal lavage and to reduce the time of appropriate intervention in pediatric patients [1].

The Rapid Ultrasound for shock and hypertension (RUSH) protocol addresses hemodynamic compromise through a conceptualization using “the pump”, “the tank” and “the pipes” [41]. The RUSH protocol has not yet been defined for an application in children, however it is possible to hypothesize that the same heart and inferior vena cava evaluations can be applied to pediatric patients while the study of the “pipes” searching for an obstruction to the circulatory flow must consider the rarity of this event in children [42]. On the other hand, and application of the RUSH protocol in evaluating children must consider for example transfontanel ultrasound of the brain to detect intraventricular hemorrhage [43].

Lastly, the use of ultrasound to measure large vessel collapse and expansion in response to provocative maneuvers and major vessel diameter for the evaluation of severe dehydration is a promising application [3]. In particular, it has been demonstrated in adult patients that in rapid loss of volume due to hemorrhage or slower loss of volume due to vomiting or diarrhea etc., the ultrasound study of the inferior vena cava collapsibility can give an added value to the clinical evaluation of the patient, whereas data concerning pediatric patients are still conflicting [44].

5) ***Musculoskeletal: US in skeletal fractures and hip effusion***

#### Skeletal fractures

With the advances in ultrasound technology, US imaging of the musculoskeletal system has not only become a highly informative procedure but also a comfortable and time-efficient technique for the examiner [45]. Depending on the clinical situation and on the physician’s expertise, US imaging may be used as a primary imaging tool as it has been shown for clavicular fracture [46], fractures of the forearm and simple diaphyseal fractures of the hummers and femur [47] or in radiographically indeterminate findings and may replace follow up radiography [47].

Fractures are usually visualized with US using a high frequency linear probe that can identify millimetric interruptions in the hyperechoic line of the bone cortex [48]. In some situations, US is even more accurate than traditional radiology in the identification of lesions such as sternal and rib fractures, greenstick fractures and in those fractures, that have an important involvement of the adjacent structures and connective tissues, to identify edema and hematomas [49].

One of the most common type of fractures in children are those of the forearm, followed by the humerus and phalanges. Ultrasound guided manipulation and reduction of these fractures is recommended in literature [50] as it has been shown to reduce the need for repeated radiographs, sedation, and as it is associated with lower levels of pain and higher caregiver satisfaction [51, 52]. The ability of ultrasound to identify subperiosteal hematoma makes this procedure particularly valuable in the early recognition of possible abusive skeletal injury [53]. For example, imaging of the ribs by US can be useful in infants and suspect cases of child abuse [54]. In particular US allows each rib to be examined parallel to its long axis with concurrent palpation, unlike CT, and it is not affected by respiratory motion as in MR imaging [55]. Ultrasound may also demonstrate a significant traumatic intracranial lesion in infants with an open fontanel but it lacks sensitivity to replace CT as a primary imaging tool [56].

#### Hip effusion

One the most common challenges faced by the ED physician is to make the distinction between transient synovitis and septic arthritis in children presenting to ED with a limp. Although traditionally, hip ultrasound is performed in the radiology department, several case reports have shown that emergency physicians can use point-of-care US to identify hip effusions [57–60]. In particular, Vieira and Levy conducted a prospective study in which hip US was performed by ED physicians in 28 children with hip pain [61]. The authors found that when US was performed by ED physicians with a high confidence in US accuracy, sensitivity increased to 90% and specificity to 100%, concluding that pediatric emergency physicians are able to accurately identify hip effusions children by using POCUS.

### Procedural applications of US in Accidents & Emergencies

Pediatric emergency medicine procedures can be subdivided in 4 groups: peripheral vascular access, drainage of fluid collections and bladder catheterizations, and foreign body identification [1]. Procedural US guidance can be further divided in US-assisted or static procedures (ultrasound is used before the procedure to identify anatomic structures and ideal circumstances for the procedure) and US-guided or dynamic procedures (ultrasound and the procedure are performed simultaneously).

### Peripheral and central vascular access

While central venous cannulation is not commonly practiced by pediatricians, peripheral vascular access is generally performed in most patients in the PEDs to collect blood specimens and to provide a route for intravenous therapy [62]. In the pediatric population venipuncture and intravenous cannulation are often associated with stress, pain, crying, and can even fail due to a child’s irritability [63]. Alleviating pediatric pain experience has become a major interest in pediatric departments as the reduction of pain associated with procedures may result in diminished stress and fear of similar procedures also in the future [64].

It has been reported that the use of US guidance improves significantly the rate of successful peripheral intravenous access especially in those patients who are difficult to access, decreasing the amount of time to perform the procedure, the number of percutaneous punctures and needle redirections compared to traditional approaches such as palpation and landmark guidance [62, 65]. Infants may be particularly challenging, and US guidance has been shown to achieve success in more than 95% of cases for accessing the saphenous vein, even in children younger than 6 months [66]. In the pediatric emergency department, central venous catheter placement may be required in life threatening conditions where fluid and drug resuscitation is needed or in complex patients with poor vascular access. Ultrasound-guided pediatric CVC insertion has been shown to be superior to traditional landmark and palpation techniques [67]. The lack of fat and the fact that vessels are quite superficial allows good echografic imaging; in particular the ultrasound-guided cannulation of the brachiocephalic vein is gaining worldwide consensus for central venous access in small children, neonates, and premature infants [68].

### Abnormal fluid collections drainage, bladder catheterizations and suprapubic aspirations

US guidance may improve success and decrease complications in many procedures performed by multiple specialties, including paracentesis, thoracentesis, incision and drainage of abscesses, arthrocentesis, lumbar puncture, biopsies, and other procedures [69]. Diagnostic or therapeutic aspiration of fluid without ultrasound guidance can be difficult and can potentially injure important structures, particularly in small children [3].

Ultrasound findings of skin infections range from the non-specific finding of interstitial fluid to compressible and measurable collections of fluid that may be infected [3], in absence of blood flow within them [70]. Particularly common in the pediatric population, the visualization of abscesses by ultrasound can confirm the need for a drainage procedure and may help in decreasing repeated visits, antibiotic use, and important patient discomfort [42]. On the other hand, the visualization of cellulitis without abscess can prevent an unnecessary surgical incision [42]. In particular Adams et al. [71] demonstrated in a prospectic study that the sensitivity and specificity of point of care ultrasonography for the presence of abscess were 96 and 87% respectively, compared to the sensitivity and specificity of physical examination alone (respectively 84 and 60%). These data have been extensively confirmed in a recent a meta-analysis [72].

Urethral catheterization is quite frequently performed for a urinalysis and culture, management of acute urinary retention, and monitoring of the urine output in pediatric emergency and critical care settings [73]. In particular, if the presence of urine in the bladder is not certain before urethral catheterization, ultrasound guidance can contribute to the estimation of the amount of urine leading to increasing success rate during the first attempt in younger children [74].

Suprapubic aspiration is considered the gold standard for sterile urine collection in small children because of the low risk of contamination by fecal and skin flora compared with other methods of urine collection [75]. However repeated attempts of aspiration expose children to unnecessary pain, delay appropriate treatment and may increase the low risks of complications such as hematuria, hemorrhage and bowel perforation. According to some authors [75], the use of bladder ultrasound to assist this procedure increases success rate of suprapubic aspiration. This procedure however is not commonly used in most European pediatric wards due to its invasiveness and possible complications.

### Foreign body identification

Although they are currently the reference standard to identify ingested foreign bodies, radiographs do not identify radiolucent foreign bodies. Therefore, the absence of ingested foreign bodies on radiography can only rule out the presence of radiopaque objects, whereas ultrasound has been proven superior to radiographs in the detection of foreign bodies with low radiopacity including those made of wood, acrylic and plastic [76]. Most foreign bodies are seen as bright (hyperechoic) structures with a posterior shadowing. A careful use of ultrasound as well as detecting glass, wood or plastic foreign bodies, can also guide their removal and confirm complete removal [77]. Ultrasound has been reported to be useful for ingested esophageal, gastric and intestinal foreign bodies [78]. Furthermore, this technique has also been found to be extremely effective for identification of radiolucent subcutaneous foreign bodies [79]. With an increase in training, it is possible that emergency physicians may begin to use POCUS for identification and monitoring of ingested foreign bodies in the pediatric population.

## Conclusions

Bedside emergency ultrasound is routinely used by adult emergency physicians and in the last 10 years its application has been recognized and applied in pediatric emergency departments. POCUS in PED can be mainly divided in diagnostic and procedural applications. Literature regarding the usefulness of point-of-care ultrasound in the pediatric setting is limited and it is still not well codified in the pediatric practice. Medical literature well supports the utility of POCUS in the PED but work is still needed to define standards for the practice.

Dedicated training is required to familiarize with POCUS in pediatric emergency settings and its fields of application. False negative results are not enough to rule out a suspected diagnosis but information obtained with POCUS can generally help in the procedure of understanding clinical findings and may point towards the final diagnosis suggesting the need of ulterior investigations.

Pediatric emergency physicians are encouraged to familiarize with POCUS as it is a safe technology and can be extremely helpful in performing diagnosis, managing critical situations and guiding procedures, which results in globally improving pediatric patients care.

## Abbreviations

FAST: Focused Assessment with Sonography for Trauma
PEDs: Pediatric Emergency Departments
PEMs: Pediatric emergency physicians
POCE: Point-of-care echocardiography
POCUS: Point-of-care ultrasonography
US: Ultrasound
